# Interaction of N‐acetyl‐l‐glutamate kinase with the PII signal transducer in the non‐photosynthetic alga *Polytomella parva*: Co‐evolution towards a hetero‐oligomeric enzyme

**DOI:** 10.1111/febs.14989

**Published:** 2019-07-26

**Authors:** Khaled A. Selim, Tatyana Lapina, Karl Forchhammer, Elena Ermilova

**Affiliations:** ^1^ Department of Microbiology/Organismic Interactions Interfaculty Institute of Microbiology and Infection Medicine Eberhard‐Karls‐Universität Tübingen Germany; ^2^ Biological Faculty Saint‐Petersburg State University Russia

**Keywords:** algal metabolomics, arginine biosynthesis, N‐acetyl‐l‐glutamate kinase, Nonphotosynthetic plastids, PII‐signalling, TCA/GS‐GOGAT cycles

## Abstract

During evolution, several algae and plants became heterotrophic and lost photosynthesis; however, in most cases, a nonphotosynthetic plastid was maintained. Among these organisms, the colourless alga *Polytomella parva* is a special case, as its plastid is devoid of any DNA, but is maintained for specific metabolic tasks carried out by nuclear encoded enzymes. This makes *P. parva* attractive to study molecular events underlying the transition from autotrophic to heterotrophic lifestyle. Here we characterize metabolic adaptation strategies of *P. parva* in comparison to the closely related photosynthetic alga *Chlamydomonas reinhardtii* with a focus on the role of plastid‐localized PII signalling protein. *Polytomella parva* accumulates significantly higher amounts of most TCA cycle intermediates as well as glutamate, aspartate and arginine, the latter being specific for the colourless plastid. Correlating with the altered metabolite status, the carbon/nitrogen sensory PII signalling protein and its regulatory target N‐acetyl‐l‐glutamate‐kinase (NAGK; the controlling enzyme of arginine biosynthesis) show unique features: They have co‐evolved into a stable hetero‐oligomeric complex, irrespective of effector molecules. The PII signalling protein, so far known as a transiently interacting signalling protein, appears as a permanent subunit of the enzyme NAGK. NAGK requires PII to properly sense the feedback inhibitor arginine, and moreover, PII tunes arginine‐inhibition in response to glutamine. No other PII effector molecules interfere, indicating that the PII‐NAGK system in *P. parva* has lost the ability to estimate the cellular energy and carbon status but has specialized to provide an entirely glutamine‐dependent arginine feedback control, highlighting the evolutionary plasticity of PII signalling system.

## Graphical abstract

The major target of the PII signalling protein in Archaeplastida is N‐acetyl glutamate kinase (NAGK). PII is known as a transiently interacting signalling protein. In this work, we demonstrate that NAGK co‐evolved with PII signalling protein towards a stable hetero‐oligomeric complex that tunes arginine feedback inhibition (+ve/−ve) in response to glutamine status in nonphotosynthetic alga *Polytomella parva*. This is likely to be a metabolic adaptation strategy in line with the evolutionary loss of photosynthesis in this species.

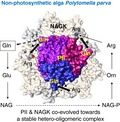

**Abbreviations**
2‐OG: 2‐oxoglutarate
Arg: arginine
*Cr*NAGK: *Chlamydomonas reinhardtii* NAGK protein
*Cr*PII: *Chlamydomonas reinhardtii* PII protein
Gln: glutamine
GOGAT: glutamate synthase
GS: glutamine synthase
NAGK: N‐acetyl‐l‐glutamate kinase
*Os*PII: *Oryza sativa* PII protein
PEP: phosphoenolpyruvate
*Ppa*NAGK: *Polytomella parva* NAGK protein
*Ppa*PII: *Polytomella parva* PII protein
SEC‐MALS: size exclusion chromatography coupled with multiangle light scattering
SPR: surface plasmon resonance spectroscopy
TCA: tricarboxylic acid cycle

## Introduction

The loss of photosynthesis is always accompanied by heterotrophic lifestyles and arose in diverse eukaryotic lineages 1. In the course of evolution, many algal species and land plants lost photosynthesis and became heterotrophic 1, 2, 3, 4, 5. Most of these nonphotosynthetic organisms still retain the plastids, which contain a small genome to carry out various nonphotosynthetic metabolic reactions 1, 5, 6. Several of these colourless algae evolved into parasites, such as the Apicomplexa lineage. *Polytomella* is a genus of colourless, free‐living unicellular nonphotosynthetic green algae, closely related to the photosynthetic green alga *Chlamydomonas reinhardtii* 1, 7, 8. Recently, the plastids of *Polytomella* spp. have been identified to be the first algae harbouring‐plastid devoid of any plastid genomes 1, while, the *Rafflesia* genus was identified to be the first parasitic plant with no recognizable plastid genome 5.

RNA‐seq analysis of *Polytomella parva* uncovered transcripts for a large set of nuclear encoded, plastid‐targeted enzymes mainly involved in carbohydrate and starch metabolism as well as amino acid and fatty acid biosynthesis 1. This implies that *P. parva* has maintained a nonphotosynthetic plastid for metabolic purposes as a specialized anabolic organelle 1, 2, 7, 8. Therefore, *P. parva* is an attractive model system for exploring the evolutionary pressure to maintain plastids in the absence photosynthesis. Up to now, the question how the primary metabolism in *Polytomella* spp., has adapted to the loss of photosynthesis has not been experimentally approached. Therefore, we started a first characterization concerning the biochemical and metabolic adaptation strategies of *P. parva* in response to different nitrogen regimes. Notably, *P. parva* was found to possess nuclear genes predicted to encode a plastid‐targeted PII signalling protein (*Ppa*PII, plastid‐targeted) and the enzymes of the ornithine/arginine biosynthesis pathway, in particular the target of PII regulation, N‐acetyl‐l‐glutamate kinase (http://www.chem.qmul.ac.uk/iubmb/enzyme/EC2/7/2/8.html) (*Ppa*NAGK, plastid‐targeted), which catalyses the commented step of arginine biosynthesis. These two proteins co‐evolved in the course of endosymbiotic generation of plastids 9 and therefore, represent a prominent test case to address issues of metabolic adaptation strategies.

The PII signalling proteins constitute a large superfamily occurring in all domains of life 10, 11. The PII proteins are trimeric in the structure and are present in almost all bacteria, in nitrogen‐fixing archaea 10, 11 and in the eukaryotic Archaeplastida domain 9, 10, 11, 12. The PII homologues (GlnB and GlnK), which contain the conserved PROSITE motifs (PTM‐site: PS00496 and C‐terminal signature: PS00638) 13, are referred as canonical PII proteins (reviewed in 9, 10, 11, 12). The PII members, which demonstrate the same trimeric architectural principle as GlnB/GlnK proteins but lack their typical PROSITE signature pattern, are termed as the PII‐like proteins 10, 14.

In contrast to the high structural conservation of PII proteins, the PII controlled targets are distinct and versatile in different phylogenetic lineages. In eukaryotes, PII homologues have only been identified and characterized in Chloroplastida (green algae and land plants), where they are nuclear encoded 15, 16, 17 and in Rhodophyta, where they are coded by the plastid genome 12, 18. In both groups of eukaryotic phototrophs, PII is localized in the plastid 15, 16, 17, 18. In cyanobacteria and plants, the PII signalling proteins were found to regulate the activity of NAGK, the controlling enzyme of arginine biosynthesis 16, 17, 18, 19, 20. In green algae and land plants, NAGK activity is controlled by the cellular glutamine (Gln) levels via glutamine‐dependent PII‐NAGK complex formation, which leads to increased enzyme activity 16, 17. In contrast to PII proteins from Chloroplastida, PII of the red alga *Porphyra purpurea* controls NAGK in a similar way as shown in cyanobacteria: PII‐NAGK complex formation is antagonized 2‐oxoglutarate (2‐OG) but independent of glutamine 18. Through complex formation with PII, NAGK gets relieved from feedback inhibition by arginine (Arg) 16, 17, 18, 19, 20, leading to enhanced activity. It appears that the biochemical features of PII‐NAGK complexes reflect the metabolic adaptations during endosymbiotic evolution 9.

The present study is the first to address metabolic adaptation strategies of the nonphotosynthetic alga *P. parva* in response to nitrogen limitation in comparison to the closely related photosynthetic alga *C. reinhardtii* by performing a relative quantification of the intracellular metabolites. To gain mechanistic insights in the metabolic specialization of the *P. parva* plastid, we studied the PII‐mediated regulation of NAGK activity, which is a key step in the control of arginine biosynthesis. Surprisingly, we found unique features not described for PII‐NAGK complexes so far. *Ppa*PII forms an unusually stable complex with *Ppa*NAGK, irrespective of effector molecules. In this complex, PII tunes arginine feedback inhibition of NAGK specifically in response to varying glutamine levels, whereas the tricarboxylic acid (TCA) cycle intermediate 2‐oxoglutarate (2‐OG) and ATP/ADP nucleotides had no regulatory effect. These data indicate that the PII‐NAGK system in this nonphotosynthetic alga evolved into a hetero‐oligomeric enzyme complex that has lost the ability to estimate the current energy and carbon status of the cells but specifically responds with high sensitivity to the arginine/glutamine level of the cells.

## Results

### Metabolomic analysis

**Figure 1:**
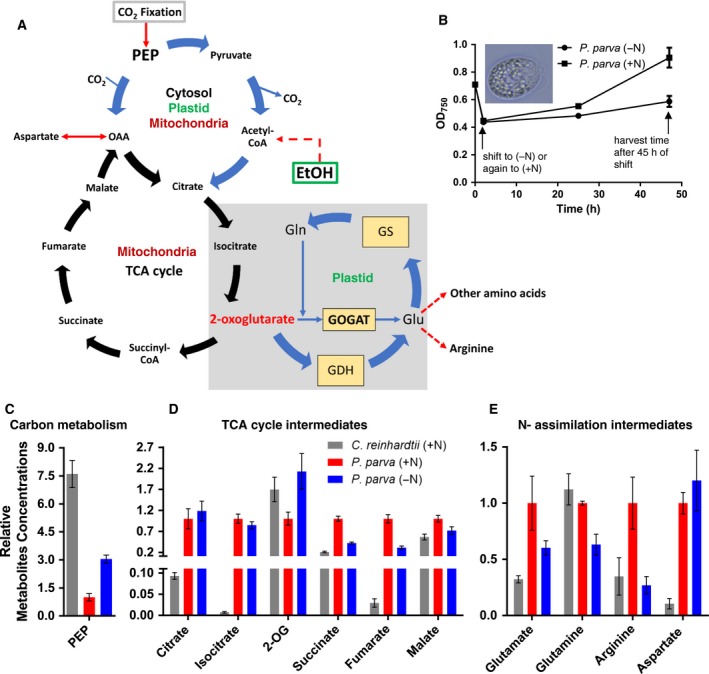
Central C‐ and N‐ metabolism in nonphotosynthetic alga *Polytomella parva* and in photosynthetic alga *Chlamydomonas reinhardtii*. (A) Inferred metabolic pathways in non‐ and photosynthetic algae *P. parva* and *C. reinhardtii*, respectively, with special reference to the TCA‐ and GS/GOGAT‐cycles. The scheme of metabolic pathway is compartmentalized in terms of mitochondrion, plastid and cytosol, according to 21. *Polytomella parva* uses ethanol as a carbon source for, while *C. reinhardtii* fixes CO_2_ or/and uses acetate as external carbon. (B) Growth of *P. parva* (inset; scale 10 μm) under N‐limited and N‐rich conditions. The arrow at the right end shows the time point (45 h) of harvesting *P. parva* for metabolite analysis. The experiment was started with an exponentially growing culture of *P. parva* under nitrogen‐rich conditions, which was collected and shifted to N‐limiting (0.375‐mm NH_4_^+^) conditions or back again to the N‐rich conditions (7.5‐mm NH_4_^+^) (arrow left). Significant metabolic alterations of (C) PEP (C‐metabolism), (D) TCA‐cycle intermediates and (E) the major amino acids of N‐assimilation reactions and GS/GOGAT‐cycle intermediates within the nonphotosynthetic algae *P. parva* cells after shift from rich‐ to low‐nitrogen conditions in comparison to the photosynthetic algae *C. reinhardtii* under rich nitrogen (7.5‐mm NH_4_^+^) condition. The metabolite concentrations are relative to *P. parva* cells under high‐nitrogen supply (normalized to 1.0, red bars) for three independent replicates, and the standard deviation (SD) is indicated by error bars.

To investigate the impact of the nonphotosynthetic lifestyle of *P. parva* on its metabolomic landscape, we applied an untargeted LC‐MS metabolomics approach to characterize the changes in the metabolomic pool sizes of *P. parva* cells under different nitrogen regimes (Fig. 1A,B) in comparison to the closely related alga *C. reinhardtii* grown under optimal mixotrophic conditions 21. We were able to identify 11 metabolites of the central carbon (C) and nitrogen (N) metabolism, mainly of TCA and GS‐GOGAT cycles (Fig. 1 and Table S1), which were significantly different between *C. reinhardtii* and *P. parva* and changed upon shift from high to low nitrogen.

Remarkably, the pools of most tricarboxylic acid (TCA) cycle intermediates (citrate, isocitrate, succinate and fumarate), except malate and 2‐OG were much higher in *P. parva* cells (Fig. 1). In striking contrast to most TCA intermediates, the levels of 2‐OG were lower in *P. parva* than in *C. reinhardtii*. This suggests an efficient nitrogen assimilatory system in *P. parva* that constantly keeps the 2‐OG levels relatively low, as compared to other TCA cycle intermediates. During nitrogen deprivation, the 2‐OG level increases in *P. parva*, as expected 22, since the consumption of 2‐OG through nitrogen assimilatory reactions is reduced. Intriguingly, the levels of phosphoenolpyruvate (PEP) show the inverse pattern than most TCA intermediates. Of note, PEP is synthesized in phototrophes from the CO_2_ fixation product 3‐phosphoglycerate (3PGA) through a few glycolytic reactions 23.

Under nitrogen‐rich conditions, *P. parva* cells accumulate around 2.9‐fold more arginine, 9.4‐fold more aspartate and 3.1‐fold more glutamate than the *C. reinhardtii* cells. This suggests again, in agreement with lower levels of 2‐OG, an efficient nitrogen assimilatory system. Nitrogen assimilation and arginine synthesis appears to take place in the colourless plastid 1, as the corresponding *C. reinhardtii* homologous enzymes, glutamine synthase (GS) and glutamate synthase (GOGAT) as well as arginine biosynthesis enzymes are plastid localized 1, 21. In contrast to the elevated levels of Glu, Asp and Arg in *P. parva*, the Gln‐levels were relatively low, which suggests a high activity of GOGAT that constantly consumes glutamine and 2‐OG to produce glutamate. The high levels of Glu correlate with high Arg levels, indicating that the controlling enzyme of the ornithine/arginine pathway, *Ppa*NAGK, should be adapted to the specific metabolic alterations in *P. parva*.

Upon shift of *P. parva* cells from nitrogen‐rich to ‐poor conditions, marked changes were mainly observed for metabolites of the TCA and GS/GOGAT cycles. The arginine, succinate and fumarate pools dropped by more than 50% (Fig. 1), whereas the malate, glutamate and glutamine (the primary nitrogen assimilation product) pools dropped by 30% to 40% (Fig. 1). The amount of aspartate increased slightly, which can be explained by diminished aspartate consumption for arginine synthesis through the argininosuccinate synthase reaction. As expected, the central TCA product 2‐OG showed a more than twofold increase upon shift to low nitrogen condition, whereas the 2‐OG precursors citrate and isocitrate did not show marked changes. The threefold increase of PEP levels under N‐limitation reflects the shift in the C:N ratio during external N‐limitation. Due to the limitation of nitrogen assimilation reactions under N‐poor conditions, the decreased utilization of glycolytic intermediates for various amino acid biosynthesis reactions could lead to increased levels of the glycolytic metabolite PEP. Overall, these metabolic changes reflect the limitation of nitrogen availability, which goes along with a slightly reduced growth of *P. parva* cells under these nitrogen‐poor conditions (Fig. 1B).

Together, the main metabolic difference between the photosynthetic alga *C. reinhardtii* and its heterotrophic relative *P. parva* concerns major metabolites of the TCA cycle, and nitrogen assimilation products glutamate, aspartate as well as arginine as a final nitrogen‐storage molecule 24. The higher levels of TCA intermediates agree with the dominance of mitochondrial metabolism in *P. parva*. The high levels of nitrogen assimilation products, in particular the nitrogen‐storage amino acid arginine, which is produced in the colourless plastid, indicates a prominent metabolic role of this organelle. To get mechanistic insights into the high Arg levels, we decided to study the interaction of the nitrogen regulatory PII protein with the key enzyme of arginine synthesis, NAGK in *P. parva*, which proved crucial in the activation of the committed step of arginine biosynthesis in plants, photosynthetic algae and cyanobacteria 9, 10, 11, 12, 16, 17, 18, 19, 20, 24, 25, 26.

### *Ppa*PII is a canonical plant PII protein

**Figure 2:**
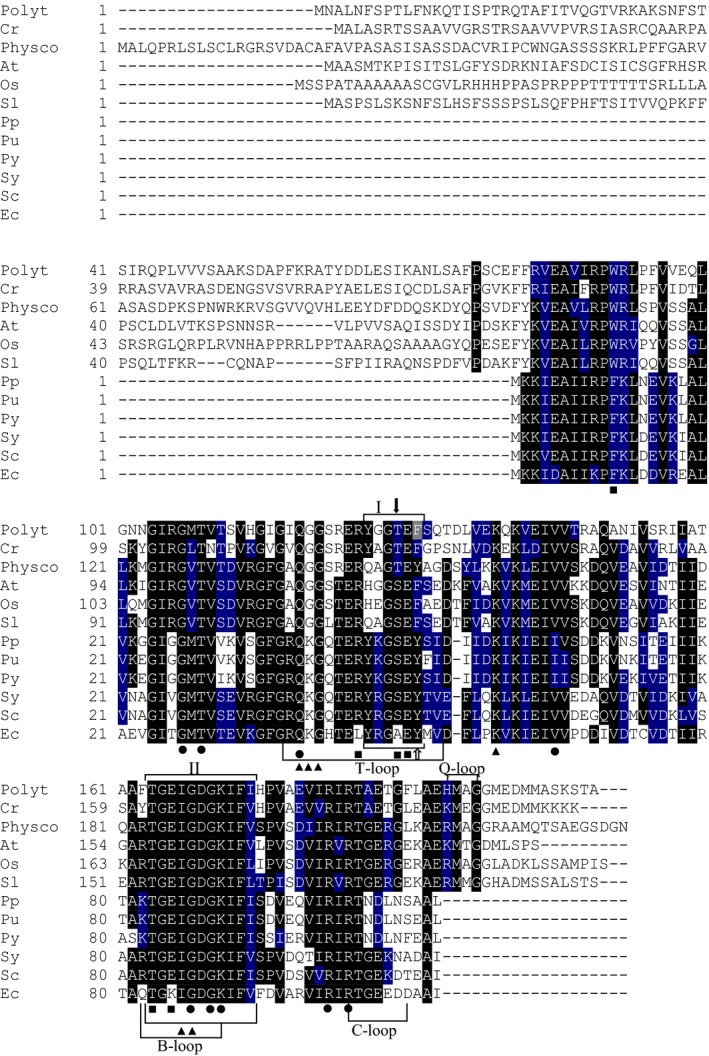
Multiple amino acid sequence alignment of PII proteins. The protein sequences were derived from NCBI database. The sequences are derived from PII polypeptides of the nonphotosynthetic alga *Polytomella parva* (Polyt), green photosynthetic alga *Chlamydomonas reinhardtii* (Cr; XP_001703658.1), land plants *Physcomitrella patens* (Physco; BAF36548.1)*, Arabidopsis thaliana* (At; NP_192099.1), *Oryza sativa Japonica* (Os; Os05g0133100) and *Solanum lycopersicum* (Sl; AAR14689.1), red algae *Porphyra purpurea* (Pp; NP_053864.1), *Porphyra umbilicalis* (Pu; AFC39923.1) and *Pyropia yezoensis* (Py; AGH27579.1), cyanobacteria *Synechococcus elongatus* PCC 7942 (Sy; P0A3F4.1), *Synechocystis* sp. PCC 6803 (Sc; CAA66127.1) and *Escherichia coli* (Ec; CAQ32926.1). All the indicated regions and residues have been characterized in previous work 17, 24, 25, 26, 27. The regions referring to T‐, B‐, C‐ and Q‐loops are indicated 17. Highlighted residues in black are invariant in at least 55% of aligned PIIs proteins. Amino acids in blue represent similar residues. Boxs I and II indicate PII signature patterns. The positions of known PIIs post‐translational modification sites: the phosphorylation site in cyanobacterial *S. elongatus* PII (S49) and the uridylation site in *E. coli* PII (Y51) are indicated by solid black and white arrows, respectively. The amino acid residues involved in binding of ATP (●), NAGK (■) and 2‐OG (▲) are indicated 24, 25, 26, 27. The alignment was done using the ClustalW program and manually refined.

**Figure 3:**
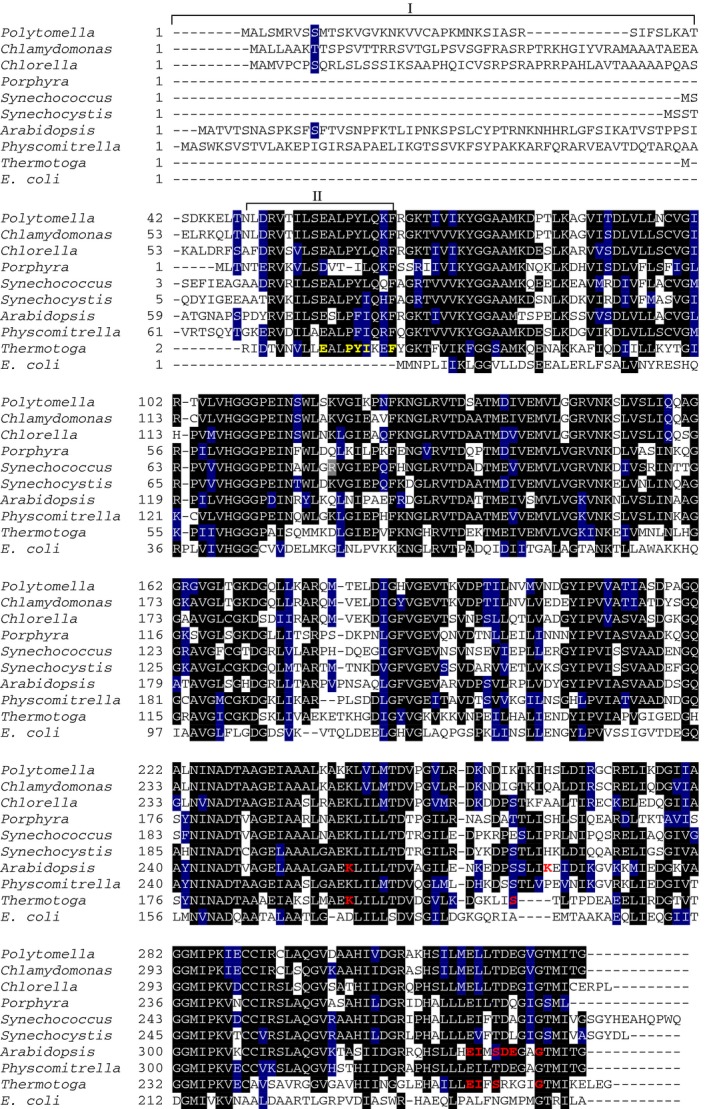
Multiple amino acid sequence alignment of NAGK proteins. The NAGK protein sequences were derived from UniProt database. The sequences are derived from NAGK polypeptides from nonphotosynthetic alga *Polytomella parva* (A6XGV3), green photosynthetic alga *Chlamydomonas reinhardtii* (A8HPI1) and *Chlorella variabilis* (E1ZQ49), land plants *Physcomitrella patens* (A0JC02) and *Arabidopsis thaliana* (Q9SCL7), red algae *Porphyra purpurea* (P69365), cyanobacteria *Synechococcus elongatus* PCC 7942 (Q6V1L5) and *Synechocystis* sp. PCC 6803 (P73326), and bacteria *Thermotoga maritima* (Q9X2A4) and *Escherichia coli* (P0A6C8). Highlighted residues in black are invariant in at least 55% of aligned NAGK proteins. Amino acids in blue represent similar residues. Box I refers to plastid‐targeting signal peptides sequence (ChloP server). Box II indicates an N‐terminal signature extension of Arg‐sensitive NAGK proteins, which is absent in Arg‐insensitive *E. coli* NAGK 28. In Box II, the previously identified signature sequence of Arg‐sensitive NAGK from *Thermotoga maritima* is highlighted in yellow, which is involved in forming the allosteric Arg binding site 28. Amino acid residues directly involved in allosteric Arg binding are highlighted in red and are deduced from known structures of NAGK: Arg complexes from *Thermotoga maritima* NAGK (PDB: http://www.rcsb.org/pdb/search/structidSearch.do?structureId=2BTY) 28 and *Arabidopsis thaliana* (PDB: http://www.rcsb.org/pdb/search/structidSearch.do?structureId=2RD5) 26. The alignment was done using the ClustalW program and manually refined.

The predicted full‐length *Ppa*PII polypeptide encoded by the *P. parva GLB1* gene consists of 209 amino acids with a calculated molecular weight of 22 745 Da and contains predicted plastid transit peptide using ChloroP 1.1 Server (amino acid residues 1‐49). As expected, the mature *Ppa*PII demonstrated the highest degree of identity with *C. reinhardtii* PII (61.78%). We performed primary sequence alignment of PII from *P. parva* with canonical PII proteins from other Archaeplastida and bacteria. The alignment of *Ppa*PII indicates extremely high local identities over two signature patterns that have been defined at the PROSITE (PS00496 and PS00638) in all canonical PII proteins (Fig. 2) 13, 27. Moreover, similar to PII homologues of Chloroplastida, *Ppa*PII protein contains the unique C‐terminal segment including the Q‐loop, which is responsible for glutamine sensing 16, 17. The alignment also showed a high degree of conservation of the functional important regions of PII proteins, including the T‐loop residues, which are involved in NAGK interactions 17, 24, 25, 26. The only noticeable variable in a contact site to NAGK concerns the tip to the T‐loop, with a Gly residue in *Ppa*PII (corresponding to Arg47 in bacterial PII proteins). The *NAG1* gene of *P. parva* encodes the full‐length NAGK polypeptide (*Ppa*NAGK) consisting of 329 amino acids with a calculated molecular weight of 34 819 Da comprising a putative N‐terminal plastid transit peptide. The *Ppa*NAGK sequence exhibits the N‐terminal signature pattern of arginine‐sensitive NAGK enzymes and the allosteric arginine‐binding site appears conserved (Fig. 3) 28. The calculated molecular weight of the predicted mature *Ppa*NAGK polypeptide is 32 587 Da.

To gain further insights into biochemical properties of *Ppa*PII and *Ppa*NAGK proteins and their mode of interaction, we prepared respective recombinant proteins. Therefore, a recombinant N‐terminal His‐tagged variant of the predicted mature *Ppa*NAGK protein without the plastid transit peptide (amino acid residues 1‐40) (the theoretical molecular mass of monomeric recombinant *Ppa*NAGK protein is 32.6 kDa), and a recombinant C‐terminal strep‐tagged version of the mature *Ppa*PII protein without the plastid transit peptide (the theoretical molecular mass of monomeric recombinant *Ppa*PII protein is 19.4 kDa) were overexpressed in *Escherichia coli* and affinity‐purified.

### *Ppa*NAGK catalytic efficiency in the absence of arginine is not influenced by PII

**Figure 4:**
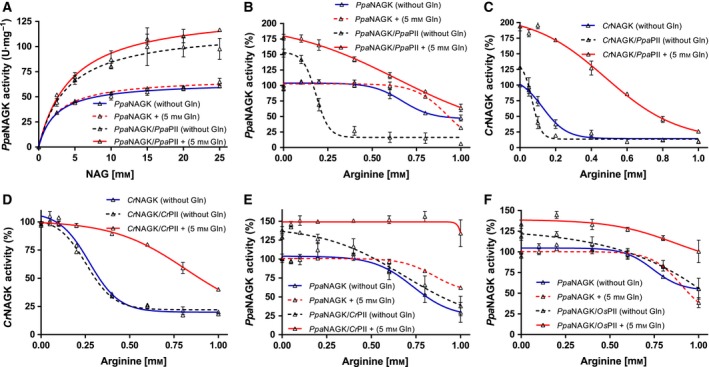
Characterization of PIIs modulated NAGK activity. (A) Catalytic activity of *Ppa* NAGK in presence or absence of *Ppa* PII and of 5‐mm Gln, as indicated. NAG was used as a variable substrate, as indicated. (B–F) Arginine feedback inhibition of NAGK enzymes in presence or absence of PII proteins, with or without 5‐mm glutamine, as indicated. (B) *Ppa* NAGK with *Ppa* PII; (C) *Cr* NAGK with *Ppa* PII; (D) *Cr* NAGK with *Cr* PII; (E) *Ppa* NAGK with *Cr* PII and (F) *Ppa* NAGK with *Os* PII. The Arg‐IC_50_ in (D) for free *Cr* NAGK (0.27 ± 0.02 mm), *Cr* PII‐*Cr* NAGK in absence of Gln (0.25 ± 0.01) and *Cr* PII‐*Cr* NAGK in presence of Gln (0.82 ± 0.09 mm) were comparable to the previously published data 17. All data were fitted using GraphPad prism program. The arginine feedback inhibition data were fitted according to a sigmoidal dose‐response curve, yielding an IC_50_ for arginine. SD as indicated by error bars, represents independent triplicate measurements.

The kinetic constants of the purified recombinant *Ppa*NAGK enzyme in the absence of the feedback inhibitor arginine exhibited an apparent *K*_m_ value for NAG of 2.35 ± 0.22 mm and a *v*_max_ of 58.1 ± 3.0 U·mg^−1^ (corresponding to a *k*_cat_ of 211.5 ± 4.1 s^−1^) (Fig. 4A). In the presence of *Ppa*PII, the apparent *K*_m_ for NAG and the specific activity substantially increased to 3.99 ± 0.62 mm and 99.1 ± 1.2 U·mg^−1^ (*k*_cat_ of 385.4 ± 16.2 s^−1^), respectively. The *Ppa*PII‐triggered changes in the kinetic parameters of *Ppa*NAGK indicate that, as in photosynthetic alga, *Ppa*PII interacts with NAGK in *P. parva*. However, the overall catalytic efficiency (*k*_cat_/*K*_m_) was very similar for free (90 × 10^3^) or *Ppa*PII‐complexed (96.5 × 10^3^ s^−1^·m ^−1^) *Ppa*NAGK. Strikingly, addition of Gln did not cause any increase in *k*_cat_/*K*_m_ catalytic efficiency (96.9 × 10^3^ s^−1^·m ^−1^), in stark contrast to the situation in *C. reinhardtii* 17, and therefore, the overall *Ppa*NAGK catalytic efficiency was not affected by *Ppa*PII, neither in the presence nor in the absence of Gln.

### Arginine sensitivity of *Ppa*NAGK activity is enhanced by PII and modulated by glutamine

As the relief from arginine inhibition by PII‐NAGK complex formation is crucial for metabolic control of arginine biosynthesis in Cyanobacteria and Chloroplastida 17, 18, 19, 20, we asked if the presence of *Ppa*PII could change the arginine inhibition profile of *Ppa*NAGK. In the absence of *Ppa*PII, feedback inhibition by arginine of *Ppa*NAGK occurred with a half maximal inhibitory concentration (IC_50_) of 0.67 ± 0.04 mm (Fig. 4B). Strikingly, addition of *Ppa*PII protein to *Ppa*NAGK enhanced arginine sensitivity of NAGK by dropping the IC_50_ for arginine by 3.7‐fold to 0.18 ± 0.01 mm. By contrast, in the presence of glutamine (5 mm), *Ppa*PII strongly relieved *Ppa*NAGK from arginine feedback inhibition. However, in the absence of *Ppa*PII, glutamine alone had no remarkable influence on NAGK activity, indicating that *Ppa*PII either enhances or reduces the arginine‐sensitivity of *Ppa*NAGK, depending on the presence of glutamine (Fig. 4B).

To investigate further whether the increased sensitivity of the *Ppa*PII‐*Ppa*NAGK complex towards Arg, as compared to *Ppa*NAGK alone, is due to properties of *Ppa*PII or of *Ppa*NAGK, we performed heterologous enzymatic assays using the respective *C. reinhardtii* proteins (*Cr*NAGK and *Cr*PII). Of note, the *Cr*NAGK protein is inherently more sensitive towards arginine than *Ppa*NAGK 17. Furthermore, we tested the PII protein from rice plant *Oryza sativa* (*Os*PII) over *Ppa*NAGK.

Strikingly, the addition of *Ppa*PII protein to *Cr*NAGK further increased the arginine‐sensitivity of *Cr*NAGK: The IC_50_ for arginine dropped from 0.12 ± 0.03 in the absence of *Ppa*PII to 0.07 ± 0.01 in presence of *Ppa*PII (Fig. 4C). However, when 5‐mm glutamine was added to the assay, *Ppa*PII behaved as shown previously for *Cr*PII (Fig. 4D) 17, strongly relieving arginine feedback inhibition, as evidenced by the fourfold increase of the IC_50_ for arginine to 0.48 ± 0.04 mm (Fig. 4C). In contrast to *Ppa*PII, the *Cr*PII and *Os*PII proteins did not raise the arginine sensitivity of the *Ppa*NAGK in the absence of Gln (Fig. 4E,F). But contrary, the *Cr*PII and *Os*PII proteins slightly enhanced *Ppa*NAGK activity at low concentrations of Arg (up to 0.5 mm). At high arginine concentrations (in the absence of Gln), *Ppa*NAGK activity dropped in the absence (IC_50_ of 0.67 mm) or presence of the heterologous PII proteins (corresponding to IC_50_ values of 0.68 ± 0.15 with *Cr*PII and 1.5 mm with *Os*PII) (Fig. 4E,F). In the presence of 5‐mm glutamine, *Cr*PII and *Os*PII proteins relieved arginine feedback inhibition of *Ppa*NAGK, as expected 17 with IC_50_ values of 1.2 and 2.3 mm, respectively. Together, these results showed that the *Ppa*PII‐mediated enhancement of arginine‐sensitivity of NAGKs is an intrinsic property of *Ppa*PII, which it can deploy in heterologous assays with other NAGK enzymes.

**Figure 5:**
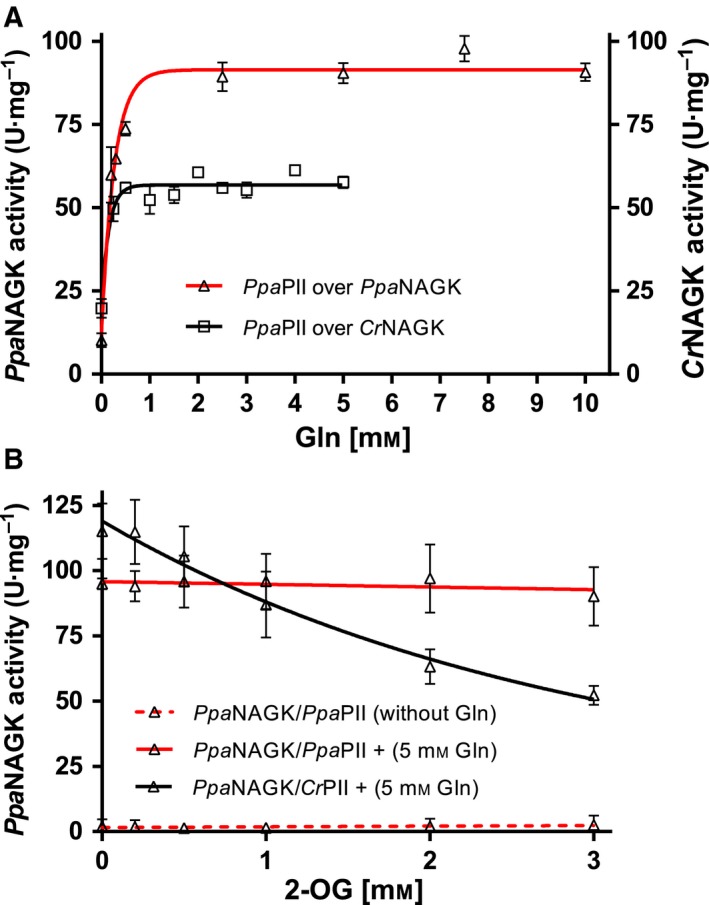
Effect of glutamine and 2‐OG on PII‐mediated NAGKs activation. (A) Glutamine‐dependent activation of arginine‐inhibited *Ppa* NAGK or arginine‐inhibited *Cr* NAGK by *Ppa* PII, as indicated. (B) Effect of 2‐OG on *Ppa* PII and *Cr* PII proteins in the presence of 5‐mm glutamine on activation of arginine‐inhibited *Ppa* NAGK, as indicated. The assays were performed in presence of 0.5‐mm arginine for *Ppa* NAGK or 0.12‐mm arginine for *Cr* NAGK. Data were fitted using a graphpad prism, yielding an EC_50_ for Gln and an IC_50_ for 2‐OG. SD as indicated by error bars, represents triplicate independent measurements.

Because glutamine increases the activity of the *Ppa*PII*‐*NAGKs complex in the presence of arginine (Fig. 4B,C), we next tested the activation of arginine‐inhibited *Ppa*PII‐*Ppa*NAGK complex (with 0.5‐mm Arg) by glutamine in a concentration‐dependent manner (Fig. 5A). The half‐maximal effective concentration (EC_50_) of glutamine for activation of the *Ppa*PII‐*Ppa*NAGK complex was determined to be 1.8 mm. A similar value was obtained for glutamine‐dependent activation of *Cr*NAGK by *Ppa*PII, with a glutamine EC_50_ of 1.1 mm (Fig. 5A). By comparison, the EC_50_ of glutamine for stimulation of *Cr*NAGK activity by *Chlamydomonas Cr*PII or *Chlorella variabilis* PII (*Cv*PII) proteins were 2.4 ± 0.8 mm 17 or 6.5 ± 1.1 mm 16, respectively. Moreover, the microalga *Myrmecia incisa* PII (*Mi*PII) also required high concentrations of Gln (3‐12 mm) to relive arginine feedback inhibited *Mi*NAGK 29 and the activation of *Arabidopsis thaliana* NAGK (*At*NAGK) by *Physcomitrella patens* PII or rice *Os*PII required also high concentrations of Gln with EC_50_ of 6.6 mm and 9.2 mm, respectively 17. This suggests that *Ppa*PII has evolved to sense lower glutamine concentrations than the other so‐far studied plant PII proteins.

### *Ppa*PII protein lacks the response to 2‐oxoglutarate

Most PII proteins were found to sense 2‐OG as the principle effector molecule in a synergistic binding reaction with ATP 10, 18, 22, 27. Recent studies have identified PII proteins from some Chloroplastida that lack 2‐OG responses 18. Therefore, we assessed the effect of 2‐OG on the modulation of *Ppa*NAGK activity by *Ppa*PII. As shown in Fig. 5B, addition of 2‐OG to a reaction mixture containing *Ppa*PII‐*Ppa*NAGK complex together with 5‐mm Gln and 0.5‐mm Arg did not lead to inhibition of *Ppa*NAGK activity, which would be expected if the complex would dissociate. As a control, the expected response towards 2‐OG was obtained for the heterologous assay with *Cr*PII, which senses 2‐OG 17, 18 with an IC_50_ of 1.99 mm. Together, it appears that the *Ppa*PII protein does not respond to 2‐OG, unlike *Cr*PII 17, 18, but it complexes with NAGK to tune its response towards the feedback inhibitor arginine in a glutamine‐dependent manner.

### Glutamine‐independent *Ppa*PII‐*Ppa*NAGK complex formation

**Figure 6:**
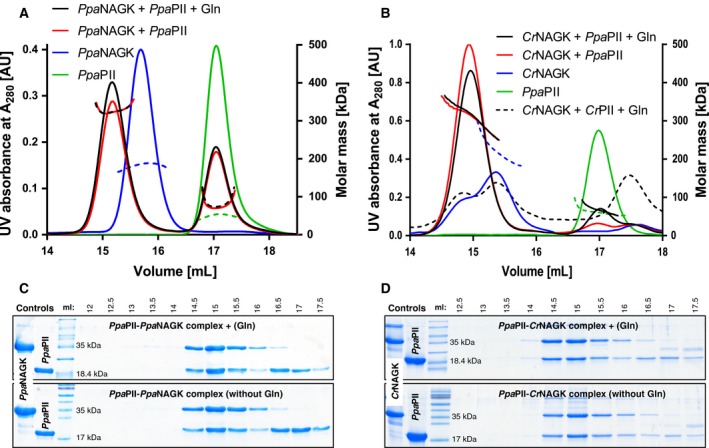
Complex formation of *Ppa* PII‐NAGKs analysed by SEC‐MALS. Gel filtration of PII‐NAGK complexes was carried out as described in Methods. SEC‐MALS profiles for (A) *Ppa* NAGK in presence or absence of *Ppa* PII and 5‐mm glutamine and (B) *Cr* NAGK in presence or absence of *Ppa* PII or of *Cr* PII with or without glutamine, as indicated. The mass of the eluted particles was determined via MALS and plotted on the right y‐axis. The protein elution profile was monitored using UV signal at 280 nm and plotted on the y‐left. (C and D) The eluted protein fractions between 12.5 to 17.5 mL corresponding to *Ppa* PII‐*Ppa* NAGK complexes as shown in (A) or for *Ppa* PII‐*Cr* NAGK complexes as shown in (B) were collected and subjected to Glycine‐SDS/PAGE, and revealed the presence of *Ppa* PII and NAGK proteins after Coomassie blue stain.

The above described enzyme tests suggested that *Ppa*PII‐*Ppa*NAGK complex formation must be different from all previously tested cases 16, 17, 18, 19, 20, 24, 25, 26, since *Ppa*PII enhances the arginine sensitivity of NAGK in the absence of glutamine. Of note, in the absence of Gln, the PII proteins from representative Chloroplastida were not able to effectively form a complex with NAGK, even in the presence Mg^2+^‐ATP 17. *Ppa*PII contains the C‐terminal Q‐loop responsible for glutamine binding (Fig. 2), that was shown previously to promote glutamine‐dependent complex formation of *Cr*PII‐*Cr*NAGK or of other plant PII proteins except *Arabidopsis* 17. To monitor any changes in molecular weight due to complex formation, we characterized PII‐NAGK complexes in the presence or absence of Gln using analytical size exclusion chromatography (SEC) coupled to multiangle light scattering (MALS). First, we determined the oligomerization state of *Ppa*PII and *Ppa*NAGK proteins. As expected, the *Ppa*PII protein eluted as a trimer and *Ppa*NAGK as a hexamer (Fig. 6A) 17, 20, 24, 25, 26. When an excess of *Ppa*PII was mixed with *Ppa*NAGK (4 : 1 monomeric concentrations), a *Ppa*PII‐*Ppa*NAGK complex was detected with a clearly detectable peak shift for the *Ppa*NAGK hexamer. In agreement with the enzymatic characterization, glutamine was not required for complex formation, nor did it induce a remarkable shift in the size of the complex (Fig. 6A).

To confirm that the *Ppa*PII protein is responsible for glutamine‐independent complex formation with NAGK, we investigated complex formation with the *Cr*NAGK protein. The *Cr*NAGK eluted as a hexamer like previously reported 17. Independent of the absence or presence of Gln, the *Ppa*PII protein was able to form a stable complex with *Cr*NAGK and both proteins co‐eluted together (Fig. 6B). In agreement, the SDS/PAGE analysis of the collected complexes’ peaks showed the presence of both *Ppa*PII with *Ppa*NAGK or with *Cr*NAGK (Fig. 6C,D). Together, the results demonstrated that the *Ppa*PII protein forms complexes with NAGKs independent of glutamine.

### Influence of different effector molecules on PII‐NAGK complex formation

To further confirm that the direct interaction between *Ppa*PII and *Ppa*NAGK is glutamine‐independent and to test the influence of the other known PII effectors molecules (ATP, ADP, 2‐OG and Gln) on the PII‐NAGK complex formation, we assessed the complex formation using surface plasmon resonance (SPR) spectroscopy. In SPR experiments, the His‐tagged NAGK protein was immobilized on a Ni‐NTA sensor chip and the strep‐tagged PII protein was injected together with or without different combinations of effectors molecules to monitor the difference in the response unites (ΔRU) due to the PII‐NAGK complex formation.

**Figure 7:**
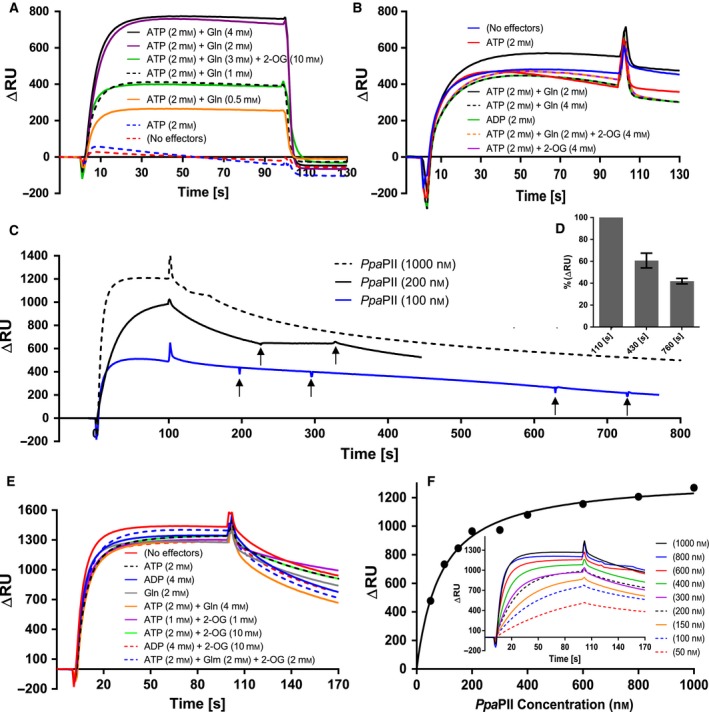
Surface plasmon resonance spectroscopy analysis of PII‐NAGK complex formation. *Cr* PII or *Ppa* PII were injected to FC2‐immobilized *Cr* NAGK or *Ppa* NAGK. (A) Strict Mg^2+^‐ATP/Gln dependency of 1000‐nm *Cr* PII binding to *Cr* NAGK. (B–F) Binding of 1000‐nm *Ppa* PII to various NAGK enzymes under various conditions. (B): Binding of *Ppa* PII to *Ppa* NAGK, as indicated. (C) Stability of the *Ppa* PII‐*Ppa* NAGK complex formed by injection of 100‐, 200‐ or 1000‐nm *Ppa* PII, as indicated, in absence of any effector molecules during SPR dissociation. The arrows indicate the injection of 2‐mm ADP, which did not affect complex stability/dissociation. (D) Dissociation of *Ppa* PII‐*Ppa* NAGK complex; shows the average of the response signals shown in (C) in form of % at *t:430s* and at *t:760s* (330s and 660s after the end of the injection, respectively). The signals at *t:110s* (10s after the end of the injection) were normalized to 100%. SD as indicated by error bars, represents triplicate independent measurements. (E) Binding of 1000‐nm *Ppa* PII to *Cr* NAGK, as indicated. (F) *K*_d_ value for binding *Ppa* PII to NAGK calculated from ∆RU at *t:100s*. The inset in (F) shows the *Ppa* PII titration (from 50 to 1000 nm) to NAGK in absence of effectors molecules, as indicated.

We showed previously that formation of the *Cr*PII‐*Cr*NAGK complex from *C. reinhardtii* was strictly Mg^2+^‐ATP and glutamine‐dependent and was not supported by ADP (Fig. 7A) 17. By contrast, *Ppa*PII was able to form a strong complex with NAGK on the SPR surface, independent of presence or absence of ADP, ATP, 2‐OG and Gln (Fig. 7B). Remarkably, the *Ppa*PII‐*Ppa*NAGK complex was extraordinary stable and dissociated very slowly in the course of the assay with an estimated *K*_*d*_ value of 93.8 ± 29.9 nm (Fig. 7C,D). The percent of *Ppa*PII‐*Ppa*NAGK complex dissociation from the sensor chip at 330 and 660 sec after the end of the injection phase was 60.7% and 41.9%, respectively (RU at 110 sec was taken as 100%) (Fig. 7D), indicating the stability of the complex. By contrast, in the case of *Cr*PII‐*Cr*NAGK, the complex dissociated spontaneously at the end of the injection as soon as it encountered a buffer devoid of Mg^2+^‐ATP and Gln (compare Fig. 7A,C).

Furthermore, we reported previously that ADP and 2‐OG negatively affected cyanobacterial PII‐NAGK interaction by promoting the dissociation of the complex, and further, the injection of 1 mm of ADP caused immediate dissociation of PII‐NAGK complexes 30. Remarkably, the *Ppa*PII‐*Ppa*NAGK complex was resistant against the injection of ADP, indicating that *Ppa*PII is unable to sense ADP (Fig. 7B,C). The 2‐OG effector also showed no antagonistic effect on *Ppa*PII‐*Ppa*NAGK complex formation (Fig. 7B) in agreement with the inability of 2‐OG to inhibit the NAGK enzymatic activity (Fig. 5B). This result resembled a previous result on *Cr*PII‐*Cr*NAGK interaction, where 2‐OG had no influence on the *Cr*PII‐*Cr*NAGK complex formation, while the 2‐OG mediated inhibition of *Cr*NAGK activity in complex with *Cr*PII‐complex appeared to occur postbinding 17.

To gain further insights in the Gln‐independent complex formation of *Ppa*PII, we repeated the previous SPR experiments using *Cr*NAGK as a binding partner. Regardless of the effector molecules added to the assay mixture (using 1‐μm *Ppa*PII protein), the *Ppa*PII protein formed a strong complex with *Cr*NAGK in an Mg^2+^‐ATP and Gln‐independent manner, and moreover, neither ADP nor 2‐OG had influence on *Ppa*PII‐*Cr*NAGK complex formation (Fig. 7E). As before, *Ppa*PII was able to bind to *Cr*NAGK without any effector molecules (*K*_d_ value of 86.3 ± 9.4 nm, Fig. 7F). Together, these experiments suggest that the Gln‐independent formation of the *Ppa*PII‐NAGKs complex is a unique feature of *Ppa*PII in comparison to other plant PII proteins that possess a functional Q‐loop, the latter requiring Gln for NAGK interaction, as shown for PII from green algae (*C. reinhardtii* and *Chlorella variabilis*) 16, 17, microalga (*Myrmecia incisa*) 29, or higher plants (*Oryza sativa* and *Physcomitrella patens*) 17.

**Figure 8:**
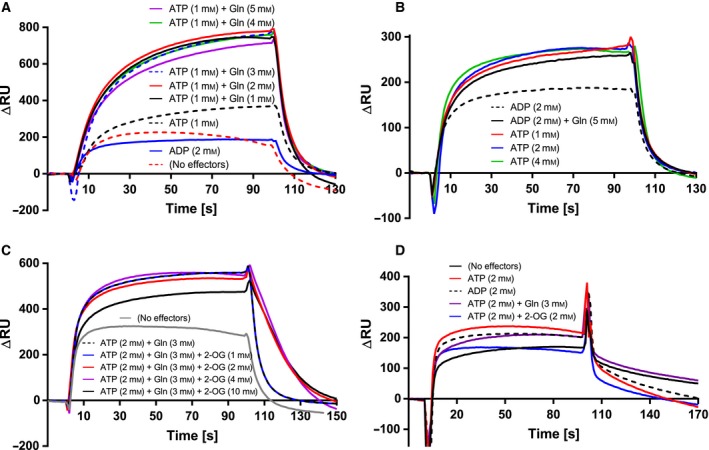
Ability of *Ppa* NAGK to bind *Cr* PII or *Os* PII in effector molecule‐independent manner. Interaction between *Ppa* NAGK and *Cr* PII or *Os* PII was analysed by SPR; 1000 nm of *Cr* PII or *Os* PII were injected to FC2‐immobilized *Ppa* NAGK. (A and B) Binding of *Cr* PII to *Ppa* NAGK, as indicated. (C) 2‐OG independent binding of *Cr* PII to *Ppa* NAGK, as indicated. (D) Binding of *Os* PII to *Ppa* NAGK, as indicated; shows no negative influence of ADP or 2‐OG on *Os* PII‐*Ppa* NAGK complex.

Finally, we asked whether the *Ppa*NAGK protein may also provide features to the glutamine‐independence of *Ppa*PII‐NAGKs complex formation. Therefore, we tested the ability of *Cr*PII and *Os*PII proteins to form heterologous complexes with *Ppa*NAGK in the absence or presence of effector molecules Mg^2+^‐ATP and Gln using SPR. As already mentioned, the formation of the *Cr*PII‐*Cr*NAGK complex is strictly dependent on Mg^2+^‐ATP and Gln (Fig. 7A), whereas Mg^2+^‐ADP did not support complex formation 17. Remarkably, *Cr*PII and *Os*PII proteins were able to bind to *Ppa*NAGK without any effector molecules (Fig. 8), indicating that *Ppa*NAGK attracts the heterologous PII proteins in a glutamine‐independent manner. Nevertheless, with *Cr*PII, the presence of Mg^2+^‐ATP alone or in combination Gln moderately or strongly enhanced the binding to *Ppa*NAGK, respectively. Interestingly, in the presence of Mg^2+^‐ADP, *Cr*PII was still able to form a weak complex with *Ppa*NAGK, similar to the absence of effector molecules (Fig. 8A), indicating that *Cr*PII lost the ability to sense ADP, confirming our previous reports 17, 18. The addition of Gln in presence of Mg^2+^‐ADP enhanced the *Cr*PII‐*Ppa*NAGK complex formation (Fig 8B), however, Gln in combination with Mg^2+^‐ATP stimulated much stronger complex *Cr*PII‐*Ppa*NAGK formation (compare Fig 8A,B). As for the homologue *Cr*PII‐*Cr*NAGK complex 17, 2‐OG did not show any influence on the *Cr*PII‐*Ppa*NAGK complex (Fig. 8C). Moreover, *Os*PII interacted with *Ppa*NAGK independent of any effector molecules (Fig. 8D). These results indicate that *Ppa*NAGK strongly influences the binding properties of various PII proteins and implies a role in the sensory properties of the entire PII‐NAGK complex.

## Discussion

Many species of Chlorophyceae, to which the *Polytomella* spp. lineages belong, including *P. parva,* contain a strongly reduced mitochondrial genome and more strikingly, the *Polytomella* spp. are the first discovered plastid‐bearing algae devoid of a plastid genome 1. Apparently, the *P. parva* plastid seems to carry out essential anabolic functions including amino acid, fatty acid, carbohydrate and lipid biosynthesis, which have not been re‐located to the cytoplasm during evolution. In *P. parva*, the genes encoding for NAGK and PII proteins were found among the plastid‐targeted/nuclear encoded genes. We hypothesized that *P. parva* must possess strong metabolic adaptations to cope with the evolutionary loss of photosynthesis.

As a consequence of the lifestyle switch towards a purely heterotrophic metabolism, the mitochondria in *P. parva* are the primary energy‐generating organelles through their respiratory activity, whereas in *C. reinhardtii*, their respiratory function is mainly limited to dark periods. In the organotrophic lifestyle of *P. parva*, it uses ethanol as a carbon source and oxidatively metabolizes it by mitochondrial activity for energy release. In agreement with the prominent role of mitochondrial metabolism, the levels of most of the TCA cycle intermediates in *P. parva* are strongly increased as compared to *C. reinhardtii*. By contrast, in *C. reinhardtii,* the elevated PEP pool (as compared to *P. parva)* agrees with a flow of carbon from CO_2_ fixation into lower glycolysis 31, to provide the cells with precursors for most anabolic pathways. Whereas, in the heterotrophic *P. parva*, PEP has to be synthesized via gluconeogenetic reactions starting from the carbon source ethanol, which can explain the 7.6‐fold decreased level of PEP in *P. parva* (Fig. 1).

Under conditions of nitrogen excess, the levels of the amino acids arginine, aspartate and glutamate are clearly elevated in *P. parva* compared with *C. reinhardtii* (Fig. 1), which suggests fast nitrogen‐assimilation reactions in *P. parva*. The GS/GOGAT cycle is the primary route for nitrogen assimilation, suggesting that this reaction cycle should be highly active in *P. parva*, in agreement with the localization of GS and GOGAT enzymes in the *P. parva* plastid 1 and the fast growth rate of *P. parva* with generation time of ~ 4.7 h at 25 °C 32. As compared to *C. reinhardtii*, the level of 2‐OG is relatively the lowest of all TCA cycle metabolites in *P. parva*. This agrees with an efficient GOGAT reaction, constantly depleting the 2‐OG pool. Since this reaction also consumes glutamine, we also find relatively lower levels of glutamine than of glutamate, aspartate or arginine. The highly active nitrogen assimilation activity results not only in elevated glutamate levels but also in high arginine levels. The controlling enzyme of the arginine synthesis pathway, NAGK, therefore, needs to be highly active. As shown here, the control of NAGK by the PII signalling protein shows unique features, which probably result from the evolutionary pressure of a nonphotosynthetic environment with a restrained adenylate energy charge to maintain NAGK at high activity.

The Arg sensitivity profile of free *Ppa*NAGK (Arg‐IC_50_ of 0.67 mm) is intermediate between the more sensitive *Chlamydomonas Cr*NAGK (Arg‐IC_50_ of 0.11 mm) 17, and the low‐sensitive NAGKs from *Arabidopsis At*NAGK (Arg‐IC_50_ of 1.0 mm) 19 or *Chlorella Cv*NAGK (Arg‐IC_50_ of 1.2 mm) 16. Thus, the higher levels of arginine required to inhibit *Ppa*NAGK in comparison to *Cr*NAGK is in good agreement with the observed higher levels of Arg production in *P. parva* (Fig. 1). Multiple sequence alignment (Fig. 3) of NAGK sequences shows that all residues participating in the allosteric Arg binding site in *At*NAGK (PDB: http://www.rcsb.org/pdb/search/structidSearch.do?structureId=2RD5) 26 are perfectly conserved in the other plant NAGKs, providing no clue to the different sensitivity towards Arg. Structural analysis of the *Ppa*PII‐*Ppa*NAGK complex in the absence or presence of arginine is required for a mechanistic explanation. In any case, in complex with its cognate PII protein, Arg‐sensitivity of *Ppa*NAGK (Arg‐IC_50_ of 0.18 mm) is similar to that of free *Cr*NAGK 17.

Unlike all other PII‐NAGK couples from cyanobacteria and plants investigated so far, the *Ppa*PII‐*Ppa*NAGK complex associates in an almost irreversible manner. The effector molecules ADP or Mg^2+^‐ATP/2‐OG, which cause efficient dissociation of the complex, are ineffective in the case of the *P. parva* proteins. Even more strikingly, the complex forms in a completely glutamine‐independent manner, although the glutamine‐sensing C‐terminal extension, the Q‐loop 17 is perfectly conserved in *Ppa*PII (Fig. 2). Since the amino acid sequences of PII and NAGK are highly conserved between *P. parva* and *C. reinhardtii* (with 61.7% and 84.5% identity, respectively), these unique features were unexpected. A few amino acid substitutions in PII may be sufficient to change the transient PII‐NAGK complex into a stable hetero‐oligomeric enzyme complex. In this respect, one residue at the tip of the T‐loop may be of particular importance: This residue, which corresponds to R47 in bacterial PII proteins, has been shown to be of key function for PII‐AmtB interaction 33. Moreover, mutation of the R47 residue in a cyanobacterial PII protein to Ala strongly reduced the affinity of *Synechococcus* PII to NAGK 25. In contrast to cyanobacteria and red algae, where this Arg residue is highly conserved, it is replaced by Ala or Glu, in many PII proteins from Chlorophyta, or in the case of *Arabidopsis* and *P. parva*, it is replaced by Gly. Conspicuously, both, *A. thaliana* and *P. parva*, bind NAGK independent of Gln, with the difference that *P. parva* nevertheless senses Gln, but *A. thaliana* PII does not, due to a truncation in its Q‐loop segment 17.

In *P. parva,* the PII protein has turned into a stably attached regulatory subunit of NAGK. The very high *in vitro* stability of the complex suggests that these proteins are co‐evolved towards the formation of a stable hetero‐oligomeric complex in an effector molecule‐independent manner, and probably are always complexed *in vivo*. In this complex, the Gln binding site resides in the PII subunit and NAGK exhibits the allosteric Arg site. Importantly, the entire complex is required for sensitive reaction of NAGK towards Arg. This requires that both *Ppa*PII and *Ppa*NAGK proteins are constantly expressed and co‐localized in the nonphotosynthetic plastid, which still needs to be experimentally proven. However, in support of this assumption, both proteins possess a plastid signal peptide at their N‐termini (amino acid residues 1‐49 for *Ppa*PII and 1‐21 for *Ppa*NAGK) (Fig. 3). Furthermore, in *C. reinhardtii* and in the microalga *Myrmecia incisa*, NAGK and PII proteins were already found to be plastid localized 16, 29, 34.

The regulatory effects of the effector molecules Gln and Arg occur in the *Ppa*PII‐*Ppa*NAGK complex at the postbinding stage. At low Gln‐levels (corresponding to N‐poor conditions), the complex is highly sensitive towards arginine. This indicates that in complex with *Ppa*PII, *Ppa*NAGK adopts a conformation that has high affinity for the allosteric feedback inhibitor arginine. By contrast, when Gln binds to the complex (under N‐rich conditions), *Ppa*PII strongly relieves *Ppa*NAGK from arginine feedback inhibition, indicating that glutamine, through binding to the C‐terminal Q‐loop, imposes a conformational change on the entire complex that counteracts the feedback inhibitory effect of arginine, like previously shown for PII‐NAGK complexes from oxygenic phototrophs 16, 17, 29. The half‐maximal effective concentration (EC_50_) of glutamine to stimulate *Ppa*PII‐NAGKs activity in presence of arginine is ~ 50% lower than the EC_50_ for *Cr*PII, showing that the *Ppa*PII protein has evolved to allow enhanced *Ppa*NAGK activity at lower glutamine concentrations. Therefore, we speculate that the default mode of the PII‐NAGK system in *P. parva* is a high arginine production through the extremely active *Ppa*PII‐*Ppa*NAGK complex, unless there is a severe nitrogen (Gln) limitation.

Analysis of the heterologous complexes formed between *P. parva* proteins and plant PII or *Cr*NAGK proteins allowed us to conclude that in *P. parva*, both partner proteins have co‐evolved towards a stable complex, with both proteins contributing to the enhanced complex stability. *Ppa*NAGK showed avid binding of *Cr*PII regardless of glutamine and of effector molecules that usually dissociate PII‐NAGK complexes (Mg^2+^‐ATP/2‐OG or ADP). Conversely, *Ppa*PII is prone to bind to NAGK proteins irrespective of effector molecules, as demonstrated by the effector molecule‐independent binding of *Ppa*PII to *Cr*NAGK (which usually only accepts glutamine‐ligated *Cr*PII as partner). This shows that the *Ppa*PII protein has evolved to exclusively sense the glutamine level in a very sensitive manner. The loss of sensing the ATP/ADP ratio and of 2‐OG might be attributed to the loss of photosynthetic activity in the plastid with consequent metabolic changes. Recently we found that the PII protein from the moss *Physcomitrella patens* 18 has also lost the ability to sense ADP and 2‐OG. This suggests that the detailed sensing properties of the PII proteins can easily be adjusted to the regulatory need of the respective metabolic situation in an organism.

Collectively, our finding extends the knowledge of PII signalling in plants. Apparently, it seems that during the evolution of Chlorophyta, the PII proteins diverged in their properties, becoming very heterogeneous with respect to 2‐OG and to ADP binding and towards complex formation with NAGK. *P. parva* is an extreme case, where the PII protein specialized its function towards a glutamine‐regulated subunit of the key enzyme of the arginine pathway NAGK. Possibly, other targets of PII regulation might have been lost during the reductive evolution of the nonphotosynthetic organelle, allowing PII to exclusively focus on NAGK regulation. It would be interesting in future to investigate PII‐NAGK systems in other secondary nonphotosynthetic organisms, to reveal if the unique feature of the PII‐NAGK complex in *P. parva* is related to the loss of photosynthesis during evolution.

## Materials and methods

### Strains and cultivation conditions

The whole cloning procedure was performed in *E. coli* NEB 10‐beta, while protein expression and purification were done using *E. coli* LEMO‐21(DE3) and PII‐deficient *E. coli* RB9060 35 in LB medium. The *Polytomella parva* SAG 63‐3 culture was obtained kindly from the algal culture collection (SAG‐Göttingen University, Germany) as an environmental nonaxenic culture. The culture was excessively treated with antibiotics until we were able to isolate a clean axenic culture of *P. parva* SAG 63‐3 (Fig. 1B). *Polytomella parva* was cultivated in REP media containing 40‐mm EtOH as a carbon source and 7.5‐mm NH_4_Cl as a nitrogen source, pH 4.0 36 at 22 °C under day/night cycles. The wild‐type *Chlamydomonas reinhardtii* CC‐125 mt+ [137c] was kindly obtained from Erik Schäffer lab. (ZMBP, Tübingen University), and cultivated in tris‐acetate‐phosphate (TAP) medium containing 7.5‐mm NH_4_Cl 37 under day/night cycles at 22 °C.

To induce nitrogen deprivation, an exponentially growing culture of *P. parva* under nitrogen‐rich condition (7.5‐mm NH_4_Cl) was harvested, washed twice in nitrogen‐free media, then suspended in fresh media, and divided into two subcultures. One subculture was re‐inoculated again into nitrogen‐rich (7.5‐mm NH_4_Cl) condition, while the other half was re‐inoculated into nitrogen‐limiting (0.375‐mm NH_4_Cl) condition. After 45 h, the *P. parva* cultures were harvested to determine the intracellular metabolites using LC‐MS, in comparison to standard growing culture of *C. reinhardtii* under nitrogen‐rich condition (7.5‐mm NH_4_Cl).

### Metabolite extraction and quantification

For quantification the intracellular metabolites of 50 mL of exponentially growing cells under the day cycle of *P. parva* under different nitrogen regimes (excess nitrogen of 7.5‐mm NH_4_Cl or poor nitrogen of 0.375‐mm NH_4_Cl), and of *C. reinhardtii* under nitrogen‐rich condition (7.5‐mm NH_4_Cl) were shock‐cooled in ice for 5 min, then rapidly harvested by centrifugation at 4 °C. After discarding of liquid media, the cell pellets were immediately frozen in liquid nitrogen. Metabolite extraction and quantification was done according to 38. Briefly, the cells were lyophilized followed by an extraction of the metabolites using a Retsch ball mill (two cycles, 30 s each). Extraction was done twice using 400 μL of 80% methanol containing 0.1% formic acid followed by a second extraction step with 400 μL of 20% methanol also containing 0.1% formic acid. The extracted metabolites were combined and concentrated in a Speed‐Vac, then dissolved in 150 μL of 20% methanol containing 0.1% formic acid (HPLC‐grade). LC/MS‐analyses were done on a Waters UPLC‐SynaptG2 LC/MS system. Chromatography was carried out on a 2.1 × 100 mm, 1.8‐μm Waters Acquity HSST3 column. For separation, a 10‐min gradient from 99% water to 99% methanol (both solvents with 0.1% formic acid) was used. The mass spectrometer was operated in ESI negative and positive mode and scanned from 50 to 2000 m/z with a scan rate of 0.5 s. For the determination of peak areas, extracted ion chromatograms were generated and integrated. The quantification of the intracellular metabolites was normalized to cell‐dry weight.

### Cloning of *Ppa*PII and *Ppa*NAGK‐like proteins

The sequences for *Ppa*NAGK homologue and *Ppa*PII were derived from iMicrobe database under project ID MMETSP0052 (https://www.imicrobe.us/#/projects/104) with sequence ID: for *Ppa*NAGK (MMETSP0052_2‐20121109|9957_1) and for *Ppa*PII (MMETSP0052_2‐20121109|12411_1). Gene Blocks, with optimized codon usage for cloning and expression into *E. coli*, encoding for amino acid sequences of mature *Ppa*NAGK and *Ppa*PII genes without plastid signal peptides, were synthesized by IDT, USA. The first Gene Block fragment for the amino acid sequence of the *Ppa*NAGK was derived from a DNA sequence starting with the 41st amino acid (TSDKK); the gene was amplified using the forward primer 5′‐TCATCATCATCACAGCAGCGGCCTGGTGCCGCGCGGCAGC‐3′ and the reverse primer 5′‐TATGCTCGAGGATCCGGCTGCTAACAAAGCCCGAAAGGAA‐3′. The second Gene Block for the DNA sequence of *Ppa*PII was derived from the amino acid sequence starting with the 50th amino acid (SAAKS) and was amplified with the forward primer 5′‐AATAGTTCGACAAAAATCTAGATAACGAGGGCAAAAAATG‐3′ and the reverse primer 5′‐CTGCAGGGGGACCATGGTCTCAGCGCTTGGAGCCACCCGC‐3′. Using Gibson assembly, the Gene Blocks for *Ppa*NAGK and *Ppa*PII were cloned directly into NdeI‐digested pET15b vector (Novagen, Darmstadt, Germany) and BsaI‐digested pASK‐IBA3 vector (IBA, Munich, Germany), respectively, as described previously 39. The plastid signal peptides were determined using a ChloroP 1.1 Server (http://www.cbs.dtu.dk/services/ChloroP/) 40, 41.

### Expression and purification of *Ppa*NAGK, *Cr*NAGK, *Ppa*PII, *Cr*PII and *Os*PII proteins

The overexpression of the recombinant N‐terminal fused His_6_‐tagged *Ppa*NAGK and *Cr*NAGK was performed in *E. coli* LEMO‐21(DE3) and the proteins were affinity purified on a Ni‐NTA columns according to 18, 42. Overexpression of the recombinant C‐terminal fused strep‐tagged PII proteins (*Ppa*PII, *Cr*PII and *Os*PII) were performed in PII‐deficient *E. coli* RB9060 35 and the proteins were affinity purified on a Strep‐Tactin II column according to 14, 20.

### Coupled NAGK activity assay

The activity of NAGK was assessed using a coupled enzyme assay in which the production of ADP after the consumption of ATP for phosphorylation of NAG was associated with the oxidation of NADH by pyruvate kinase and lactate dehydrogenase as described previously 18, 19. The standard reaction mixture consisted of 50‐mm imidazole pH 7.5, 50‐mm KCl, 20‐mm MgCl_2_, 0.4‐mm NADH, 1‐mm phosphoenolpyruvate, 5‐mm ATP, 0.5‐mm DTT, 11‐U lactate dehydrogenase, 15‐U pyruvate kinase and 50‐mm NAG and the reaction was started by the addition of 1.5‐μg NAGK. When necessary, PII protein was added to the reaction mix in equimolar concentration. When needed, the effector molecules 2‐OG, Gln and Arg were added to the reaction mixtures at concentrations as indicated. The oxidation of NADH was measured at 340 nm for 10 min with a SPECORD‐spectrophotometer (model‐210 PLUS, Analytik Jena AG). One molecule oxidation of NADH is proportional to one molecule phosphorylation of NAG. One unit of NAGK catalyses the conversion of 1 μmol of NAG min^−1^, calculated with the molar absorption coefficient of NADH of 6178 L mol^−1^·cm^−1^ at 340 nm. Means of triplicate experimental determinations are shown with a standard deviation of less than 5%. The enzymatic constants *K*_m_, *k*_cat_, IC_50_ and EC_50_ were calculated from the velocity slopes using the graphpad prism software program (GraphPad Software, San Diego, CA, USA).

### Surface plasmon resonance spectroscopy analysis (SPR spectroscopy)

SPR experiments were done at 25 °C using a BIAcore‐X biosensor system (Biacore AB, Uppsala, Sweden) in HBS buffer (10‐mm HEPES, 150‐mm NaCl, 2‐mm MgCl_2_ and 0.005% NP‐40, pH 7.5) with a flowrate of 15‐μL·min^−1^, as described previously 17, 42. The recombinant His_6_‐tagged NAGKs (*Ppa*NAGK and *Cr*NAGK) proteins were immobilized on the flow cell (FC2) of the Ni^2+^‐loaded NTA‐biosensor chip. NAGKs in HBS buffer were injected (50 μL) until a saturation of NTA‐biosensor chip by a signal of ~ 3000‐4000 resonance units (RU), which corresponds to a surface concentration change of 3‐4 (ng·mm^−2^). To evaluate the effect of the effector molecules on the PII‐NAGK complex formation for the binding of PII (*Ppa*PII, *Cr*PII and *Os*PII) proteins to the immobilized His6‐tagged NAGKs, the strep‐tagged PII proteins (100‐1000 nm) as indicated in HBS buffer, were incubated in ice for 5 min with or without different combinations of the effector molecules (as indicated). PII proteins (25 μL) were injected as an analyte into both FC1 (control for unspecific binding of PII to the sensor chip) and FC2 (immobilized NAGKs) on the sensor chip. The specific binding of PIIs to NAGKs was recorded as the difference in the response signal of FC2‐FC1 (∆RU). *Cr*PII protein dissociates immediately after the end of the injection, making immobilized NAGKs ready for the next injection. By contrast, the *Ppa*PII protein formed a strong complex with NAGK and dissociates very slowly over the time. Therefore, to refresh the NTA sensor chip for another assay, 25 μL of 1‐M imidazole pH 7.0 was injected to remove the immobilized NAGKs. To regenerate the NTA sensor chip, 50 μL of 0.4‐M EDTA pH 7.5 was injected and subsequently, the sensor chip was reloaded with Ni^2+^ and fresh NAGKs as described. The regeneration procedure was done when the response of PII binding to the immobilized NAGK started to decrease.

### Size exclusion chromatography and multiangle light scattering analysis

Analytical size exclusion chromatography was carried out as described previously 43, 44 on a Micro‐Äkta purifier system equipped with Superose 6 Increase 10/300 GL column (GE Healthcare, Freiburg, Germany). The Superose column was coupled to a triple‐angle light scattering (MALS) detector (MiniDAWN^™^ TREOS^®^ system; Wyatt Technology Corp., CA, USA) and a refractometer (Optilab T‐rEX, Wyatt). The column was calibrated using standard proteins: thyroglobulin (670 kDa), ferritin (440 kDa), globulin (158 kDa), conalbumin (75 kDa), ovalbumin (44 kDa), carbonic anhydrase (29 kDa), RNase (13.7 kDa) (Bio‐Rad gel filtration standard, GE Healthcare LMW gel filtration calibration kit). Bovine serum albumin (BSA) was used to calibrate and validate the MALS analysis. The running buffer consisted of 10‐mm Tris pH 7.8, 300‐mm NaCl, 2‐mm MgCl_2_, 0.02% NaN_3_ and 2% glycerol. The samples were centrifuged for 5 min at 18 407 ***g***, and 100 μL of the supernatant were injected for analysis with a flow rate 0.5 mL·min^−1^. The resulting data were analysed with ASTRA program (Wyatt Technology, Dernbach, Germany). The elution volume was plotted against the UV signal and molecular weight profiles. The apparent molecular weights were derived from MALS data. The chromatographic elution profiles were collected (0.5‐mL fractions) and analysed by Glycine‐SDS/PAGE.

## Conflicts of interest

The authors declare no conflict of interests.

## Author contributions

EE and KF conceived and initiated the project. KAS and KF designed the experiments. KAS and TL performed experiments. KAS interpreted the results and wrote the manuscript. KAS, EE and KF commented and edited on the manuscript. All authors analysed the results and approved the final version of the manuscript.

## Supporting information


**Table S1.** List of identified metabolites by LC‐MS normalized to 1 mg of algal cell dry weight including standard deviation (SD) of three biological replicates for *Polytomella parva* (under nitrogen excess and limiting conditions) and *Chlamydomonas reinhardtii* (under nitrogen‐rich conditions).
