# Supplementary material for: Interaction of N‐acetyl‐l‐glutamate kinase with the PII signal transducer in the non‐photosynthetic alga Polytomella parva: Co‐evolution towards a hetero‐oligomeric enzyme
Source: FEBS J. 2019 Jul 26;287(3):465–82. doi: 10.1111/febs.14989 (PMC7027753; doi:10.1111/febs.14989)
Supplement: Supplementary file 1 — Table S1. List of identified metabolites by LC‐MS normalized to 1 mg of algal cell dry weight including standard deviation (SD) of three biological replicates for Polytomella parva (under nitrogen excess and limiting conditions) and Chlamydomonas reinhardtii (under nitrogen‐rich conditions). [file FEBS-287-465-s001.zip › febs14989-sup-0001-TableS1.pdf]

The FEBS Journal
www.febsjournal.org

Interaction of N-acetyl-L-glutamate kinase with the PII signal transducer in the non-photosynthetic alga *Polytomella parva*: Co-evolution towards a hetero-oligomeric enzyme

Khaled A. Selim, Tatyana Lapina, Karl Forchhammer and Elena Ermilova

DOI: 10.1111/febs.14989

Supporting Information

Article title: **Interaction of N-acetyl-L-glutamate kinase with the PII signal transducer in the non-photosynthetic alga *Polytomella parva*: Co-evolution towards a hetero-oligomeric enzyme**

Khaled A. Selim[1,*], Tatyana Lapina[2,*], Karl Forchhammer[1] and Elena Ermilova[2]

[1]Organismic Interactions Department, Interfaculty Institute of Microbiology and Infection Medicine Tübingen, Eberhard-Karls-Universität Tübingen, Auf der Morgenstelle 28, 72076 Tübingen, Germany.
[2]Biological Faculty, Saint-Petersburg State University, Universitetskaya nab. 7/9, Saint-Petersburg 199034, Russia.

Supplementary Table S1. List of identified metabolites by LC-MS normalized to 1 mg of algal cell dry weight including standard deviation (SD) of three biological replicates for *Polytomella parva* (under nitrogen excess and limiting conditions) and *Chlamydomonas reinhardtii* (under nitrogen rich conditions).

Compound/ Organism (condition)	*C. reinhardtii* (Nitrogen rich condition, 7.5 mM NH_4^+)		*P. parva* (Nitrogen rich condition, 7.5 mM NH_4^+)		*P. parva* (Nitrogen limiting condition, 0.375 mM NH_4^+)	
	**Area of MS base-peak normalized to 1 mg cell dry weight**	**SD**	**Area of MS base-peak normalized to 1 mg cell dry weight**	**SD**	**Area of MS base-peak normalized to 1 mg cell dry weight**	**SD**
Phosphoenol pyruvate (PEP)	6.04	0.58	0.79	0.16	2.42	0.17
Citrate	72.62	6.11	780.45	187.29	924.38	183.54
α-ketoglutarate (2-OG)	22.04	3.79	12.99	2.08	27.64	5.53
Succinate	111.06	9.93	531.39	33.61	224.57	12.76
Fumarate	1.75	0.59	59.56705	5.93	18.48	2.40
Malate	269.77	33.34	476.80989	37.18	342.47	43.39
Isocitrate	0.83	0.21	113.8837	12.57	96.47	9.22
Glutamate	45.58	4.51	141.31261	33.92	85.19	8.87
Aspartate	7.88	3.42	74.238451	7.11	89.25	19.99
Glutamine	2.72	0.34	2.4224576	0.05	1.53	0.22
Arginine	20.34	9.74	58.420397	13.58	15.71	4.54
